# How can we make the most of allied health personnel?

**Published:** 2020-12-31

**Authors:** Usha Kim, Dhivya Ramasamy

**Affiliations:** 1 Director - Mid-level Ophthalmic Personnel: Aravind Eye Care System, Madurai, India.; 2 Senior Faculty, LAICO: Aravind Eye Care System, Madurai, India.

**Worldwide, allied ophthalmic personnel have become the most critical resource in delivering effective eye care. It is imperative to impart relevant training, set up standard operating procedures, and deploy robust technology to help people who are blind or partially sighted.**

**Figure:**
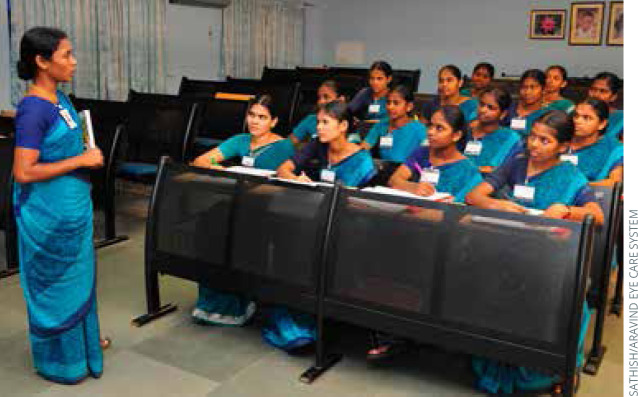
An AOP trains her team. **INDIA**

Allied ophthalmic personnel make up a vital part of the eye care workforce, as highlighted by the World Health Organization (WHO) Action Plan 2014-2019. According to WHO, opticians, ophthalmic nurses, orthoptists, ophthalmic and optometric assistants; ophthalmic and optometric technicians, vision therapists, ocularists; ophthalmic photographer/imagers, and ophthalmic administrators are collectively known as allied ophthalmic personnel. South Asia faces a severe shortage of AOPs despite having a higher prevalence of blindness, in comparison with several other regions of the world.

The role of allied ophthalmic personnel becomes crucial when there is a shortage of ophthalmologists. A well-trained person can enhance the efficiency, productivity and quality of eye care teams. Without them, eye programmes would suffer from low productivity, increased costs, and a lack of consistency in the delivery of quality eye care.

In this article, we consider ways to maximise the contribution of allied ophthalmic personnel.

## Training should be relevant

When designing a training curriculum, it is important to consider the tasks allied ophthalmic personnel may be expected to carry out (whether in the community or in a clinic) and to teach the specific competencies they require. It is equally important to focus on the aspect of attitude, alongside the skills and knowledge being imparted to trainees.

**Figure 2:**
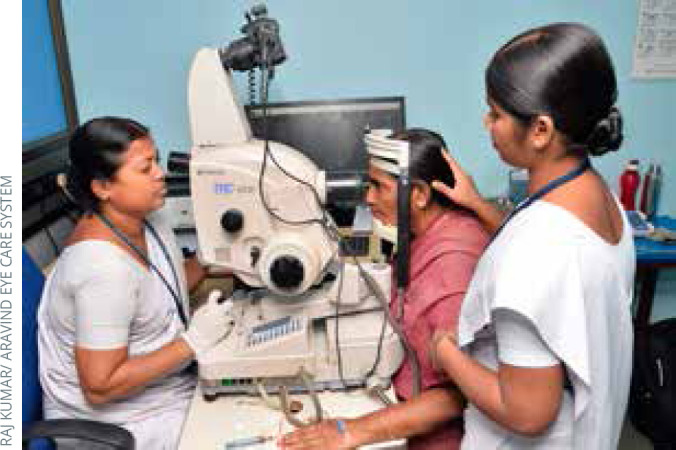
AOP staff taking fundus photographs.

We strongly advise carrying out a competency-based assessment at the end of training to ensure that trainees are ready to take up their role in the eye health workforce.

## Standard operating procedures

It is crucial to draft standard operating procedures (SOPs) to minimise inconsitencies in the eye care. The SOPs, or work instructions, provide a great deal of clarity and guidance to allied ophthalmic personnel, allowing them to work effectively and safely to provide high quality care. They also help in delivering a higher quality of care. SOPs must be drafted with the involvement of AOPs, as it ensures that the the details of each task are also standardised. Though some of these are classified as general tasks, some have to be linked explicitly with the institution.

## Part of a team

Allied ophthalmic personnel must be deployed according to need, and their skills should align with the duties they are expected to perform. The skill set of AOPs should align with the duties they are expected to perform. AOPs often work as part of a team; hence, their roles should be clearly defined within the context of their teams. At eye clinics that carry out comprehensive eye examinations, shifting the responsibility for carrying out routine tasks to allied ophthalmic personnel will help ophthalmologists work more efficiently, allowing them to be deployed more effectively. Proper execution of ‘task-shifting’ depends on the competency levels of the ophthalmic personnel.

## Ocular emergency management

Allied ophthalmic personnel should be trained to efficiently provide the first-level care for ocular emergencies, such as foreign bodies, lid lacerations, corneal injuries and chemical injuries. At the primary levels of care, where ophthalmologists are usually not available in sufficient numbers, the role of these personnel becomes vital. They should be taught when and where to refer patients that require further intervention. (See Table 1)

**Table 1:** shows different tasks and skills that could be performed by ophthalmic personnel.

In a general ophthalmology clinic:		
Trained AOP can take part in the following basics tasks:	Higher-order skills that can be acquired by trained AOPs:	Allow ophthalmologists to concentrate on these tasks:
Taking systemic and ocular history Visual acuity measurement Pupillary assessment Refraction Intraocular pressure measurement Keratometry Patient counselling Spectacle and contact lens dispensing Preparing patients and assisting for surgery Sterilisation	Preliminary examination Patient triage Visual field test Optical coherence tomography Applanation tonometry Biometry Dispensing low-vision aids Rehabilitation counselling Ophthalmic imaging	Detailed examination Diagnosis Prescription writing Minor and major procedures Surgeries

## Higher-order skills

Allied ophthalmic personnel can also be trained and deployed to perform more specialised tasks, such as dispensing low vision aids, rehabilitation, counselling caregivers of paediatric patients, and making ocular prosthesis. These functions offer new opportunities for career advancement. Training has to be intensive with sufficient practice under supervision, until candidates are certified as competent and qualified. Experienced personnel can also be deployed to supervise and train their team members.

## Equipment and instruments

It is essential to empower allied ophthalmic personnel with the instruments needed to carry out their functions well. These instruments should be in good working condition. According to WHO statistics, in developing countries and rural areas, more than 50% of the ophthalmic instruments and equipment are not functional. Dysfunctional equipment severely affects the performance of ophthalmic teams. In order to provide quality eye care services, it is essential to maintain equipment in the proper condition. With the right training, allied ophthalmic personnel can be effective in the day-to-day care and maintenance of equipment and instruments, including being able to fix common problems.

## Leveraging technology

Several new technologies are now available to help allied ophthalmic personnel to perform their tasks faster and with better accuracy. Eye institutions should familiarise themselves with new developments and invest in technologies, as required. For instance, a lensometer helps opticians determine the refractive power of a lens, faster than manual lens neutralisation methods. New technologies and mobile applications also improve speed and quality, significantly reducing training time. For example, personnel can use mobile applications such as Peek Acuity in screening. With health information management systems, mobile applications offer cheaper and portable alternatives that may be easier to use.

Today, technology can perform specific tasks that were traditionally performed by trained eye health personnel. For example, autorefractors have been shown to measure refractive errors as effectively as someone who is trained in performing refraction. These tools are simple to operate, with basic instructions, and do not require extensive training. They also play a vital role when there is a shortage of human resources for eye health.

The advent of telemedicine has brought significant gains, as it is now easier to deploy the allied personnel in remote and rural areas. Telemedicine has helped to establish an interface between patients, allied ophthalmic personnel, and ophthalmologists; thereby enhancing access to eye care. In recent years, telemedicine has also been augmented with digital imaging and artificial intelligence. Technology not only enhances the scope of services allied ophthalmic personnel can offer at the primary level, but also increases the productivity of ophthalmologists at the tertiary level.

Allied ophthalmic personnel are essential members of any eye team. It is imperative to provide them with appropriate training and clarity about their role so they can deliver high-quality eye care effectively. With the proper deployment, supported by technology, these personnel can be highly productive. In the process, it is also important to systematically identify the factors that reduce their effectiveness, and address them through continuous improvement and innovation.
